# Inflammatory landscape in Xeroderma pigmentosum patients with cutaneous melanoma

**DOI:** 10.1038/s41598-022-17928-z

**Published:** 2022-08-16

**Authors:** Asma Chikhaoui, Meriem Jones, Tadeja Režen, Melika Ben Ahmed, Chokri Naouali, Radovan Komel, Mohamed Zghal, Samir Boubaker, Sonia Abdelhak, Houda Yacoub-Youssef

**Affiliations:** 1 Laboratoire de Génomique Biomédicale Et Oncogénétique (LR16IPT05), Institut Pasteur de Tunis, Tunis, Tunisia; 2 Université Tunis El Manar, Tunis, Tunisia; 3 Département de Dermatologie, Hôpital Charles Nicolle de Tunis, Tunis, Tunisia; 4 Faculty of Medicine, Centre for Functional Genomics and Bio-Chips and Medical Centre for Molecular Biology, Institute of Biochemistry and Molecular Genetics, University of Ljubljana, Ljubljana, Slovenia; 5 Laboratoire de Transmission, Contrôle Et Immunobiologie de L’infection, LR16IPT02, Institut Pasteur de Tunis Université de Tunis El Manar I, 2092 Tunis, Tunisia

**Keywords:** Melanoma, Monocytes and macrophages, Nucleotide excision repair, Biomarkers

## Abstract

Xeroderma pigmentosum (XP) is a DNA repair disease that predisposes to early skin cancers as cutaneous melanoma. Melanoma microenvironment contains inflammatory mediators, which would be interesting biomarkers for the prognosis or for the identification of novel therapeutic targets. We used a PCR array to evaluate the transcriptional pattern of 84 inflammatory genes in melanoma tumors obtained from XP patients (XP-Mel) and in sporadic melanoma (SP-Mel) compared to healthy skin. Commonly expressed inflammatory genes were further explored via GTEx and GEPIA databases. The differentially expressed inflammatory genes in XP were compared to their expression in skin exposed to UVs, and evaluated on the basis of the overall survival outcomes of patients with melanoma. Monocyte subsets of patients with SP-Mel, XP and healthy donors were also assessed. PCR array data revealed that 34 inflammatory genes were under-expressed in XP-Mel compared to SP-Mel. Differentially expressed genes that were common in XP-Mel and SP-Mel were correlated with the transcriptomic datasets from GEPIA and GTEx and highlighted the implication of *KLK1* and *IL8* in the tumorigenesis. We showed also that in XP-Mel tumors, there was an overexpression of *KLK6* and *KLK10* genes, which seems to be associated with a bad survival rate. As for the innate immunity, we observed a decrease of intermediate monocytes in patients with SP-Mel and in XP. We highlight an alteration in the immune response in XP patients. We identified candidate biomarkers involved in the tumorigenesis, and in the survival of patients with melanoma. Intermediate monocyte’s in patients at risk could be a prognostic biomarker for melanoma outcome.

## Introduction

Melanoma is among the most lethal forms of skin cancers. Its incidence in Tunisia is relatively low (0.5–0.7 per 100,000 inhabitants per year)^1^. The risk of developing melanoma is greater 1000-fold in Xeroderma Pigmentosum (XP), a rare genetic disorder with an incidence of 1/1,000,000 in Europe^2^. This risk of cancer is greater than that observed in the general population. Indeed, more than 67% of XP patients develop a cutaneous cancer at an average age of 8 years old^3,4^. Metastatic malignant melanoma and squamous cell carcinoma are the two most common causes of death in XP patients^3–5^. In detail, XP is an autosomal recessive disease caused by a defect in one out of the 8 genes; 7 involved in the nucleotide excision repair pathway (*XPA* to *XPG*) and Variant (XP-V), that it is caused by mutations in the POLH gene, which codes for the translesion DNA polymerase^6^. All these genes greatly predisposes affected individuals to melanoma and other skin tumors in sun-exposed areas^4,7^. This rare pathology is, therefore, considered as an excellent model for studying cancer development, given the fact that, even with optimal protection against UV radiation, XP patients develop internal cancers^8^.

Decades of cancer research, supported by novel screening technologies, have revealed that tumor progression is caused by many interconnected pathways. Most cancer studies focus on studying melanoma tumors at advanced stages. However, little is known about genetic diseases predisposing to skin cancers as for the XP disease, which constitute an interesting model to study early stages of cancer development and progression. Indeed, the skin is impacted by the accumulation of genetic defects linked to poor DNA repair following repeated exposure to UVs over the years. Transcriptomic analysis for candidate genes with limited biological sampling is considered as an interesting approach in rare genetic diseases^9^, and PCR array is an example.

The mechanisms involved in melanoma cancer predisposition and development in XP patients have been mainly linked to the DNA repair defects. While, in other DNA repair syndromes that predispose to cancer such as Fanconi anemia and Bloom syndrome, severe immune alterations have been described^10,11^. In XP patients, very limited data are available regarding immune landscape^12^.

Different biological parameters, such as epidermal hyperplasia, inflammation, and oxidative stress, are important factors that contribute to the development and/or progression of skin cancers^13,14^. In particular, chronic inflammation is thought to be responsible for around 25% of all tumor types^15^. Several inflammatory mediators can cause DNA damages when it’s chronical^16^. Skin cancer has important inflammatory components contributing to each stage^17^. Inflammation is a self-limiting process, during tumorigenesis. It has been shown that alterations in its pathways can play both pro and anti-tumor effects^18^. Recently, it has been suggested that exploring the expression of different inflammatory mediators could help discover relevant biomarkers to stratify patients with cutaneous melanoma for immunotherapy and targeted therapy^19^. Although significant progress has been made in the field of melanoma immunotherapies, the identification of valuable biomarkers for monitoring melanoma prognosis or staging are limited to a few markers such as S100B protein in the serum^20^ or PDL1 who varies according to tumor infiltrating lymphocytes rate^21^.

Furthermore, the innate immunity plays an important role in preserving skin integrity against different external aggressions such as pathogen infections^22^ and UVs radiations. It regulates the reparation of DNA damage by generating inflammatory “sensor” signals such as cyclic GMP-AMP synthase (cGAS), stimulator of interferon genes (STING), the RIG-I-like receptors (RLRs) and Mitochondrial antiviral-signaling protein (MAVS) pathways, which are responsible for cytosolic DNA and RNA sensing^23,24^. Monocytes are innate immune cells, they have the role of protecting and defending the body against foreign substances, pathogens and tumor cells. In the last few years, our understanding of this population has shifted from a simple observation of a homogeneous group to a heterogeneous cluster of cells that exhibit diverse responses to different antigens. Thus, they can be classified into three subsets according to the expression of CD14 and CD16 surface markers: classical monocytes (CD14 +  + /CD16-), intermediate monocytes (CD14 +  + /CD16 +), and non-classical monocytes (CD14 + /CD16 + +)^25^. Several studies, reported that phenotypically similar monocyte sub-populations might play opposite roles in cancer development^26^. Monocyte's phenotyping in patients with melanoma was limited to two studies: the first one explored monocyte subsets in patients with melanoma skin cancer in untreated stage IV, while the second one assessed the effect of ipilimumab treatment on ex-vivo monocyte phenotypes^27,28^. Identifying monocyte subsets phenotypes in DNA repair disorders, as for XP patients who are prone to develop skin cancers at early ages, will be important in understanding the first stages of cancerogenesis.

The aims of this study are to investigate the differential expression of 84 candidate inflammatory genes in melanoma tumors (sporadic and from XP patients), and to identify the monocyte subsets, which would be associated with the early development of melanoma cancer.

## Results

### Transcriptional profile of inflammatory genes in SP-Mel and XP-Mel

Immunity landscape in cutaneous melanoma microenvironment was investigated using a pathway-focused PCR array including 84 inflammatory-related genes. Three sporadic melanoma (SP-Mel) and 3 melanoma biopsies obtained from XP patients (XP-Mel) were analyzed compared to 3 healthy skin. The relative gene expression was measured in the RNA extracted from those tumors. The selected genes encode for different inflammatory pathways related to leukotriene, complement, interferon pathways, etc.…

As a result, and due to the rarity of samples, fold change analysis *p*-value did only give a significant over-expression for *KLK6* (fold change = 5; *p*-value = 0.03) in XP-Mel group. In order to further explore the disparity in these samples, we set a threshold for the fold change and considered genes whose expression was above the two-fold as a differentially over-expressed gene and those whose expression was below 0.5 fold as a differentially under-expressed gene. As a first overview, 44 out of 84 studied genes showed altered expression in XP-Mel compared to healthy controls, while for the SP-Mel, 34 out of 84 genes were altered compared to healthy controls (Supplementary Table S1). In details comparative analysis between over and under-expressed genes in both groups suggested the existence of a common pathway via Fisher exact test (*p* value < 0.001).

We drew a Venn diagram for over and under-expressed genes from PCR array results of XP-Mel and SP-Mel tumors to delimit overlapping inflammatory gene expression. Among the examined genes, we noted in XP-Mel, the over-expression of 10 genes, while in SP-Mel group 25 genes were over expressed. Among theses over expressed genes, four were in common.

**Figure 1:**
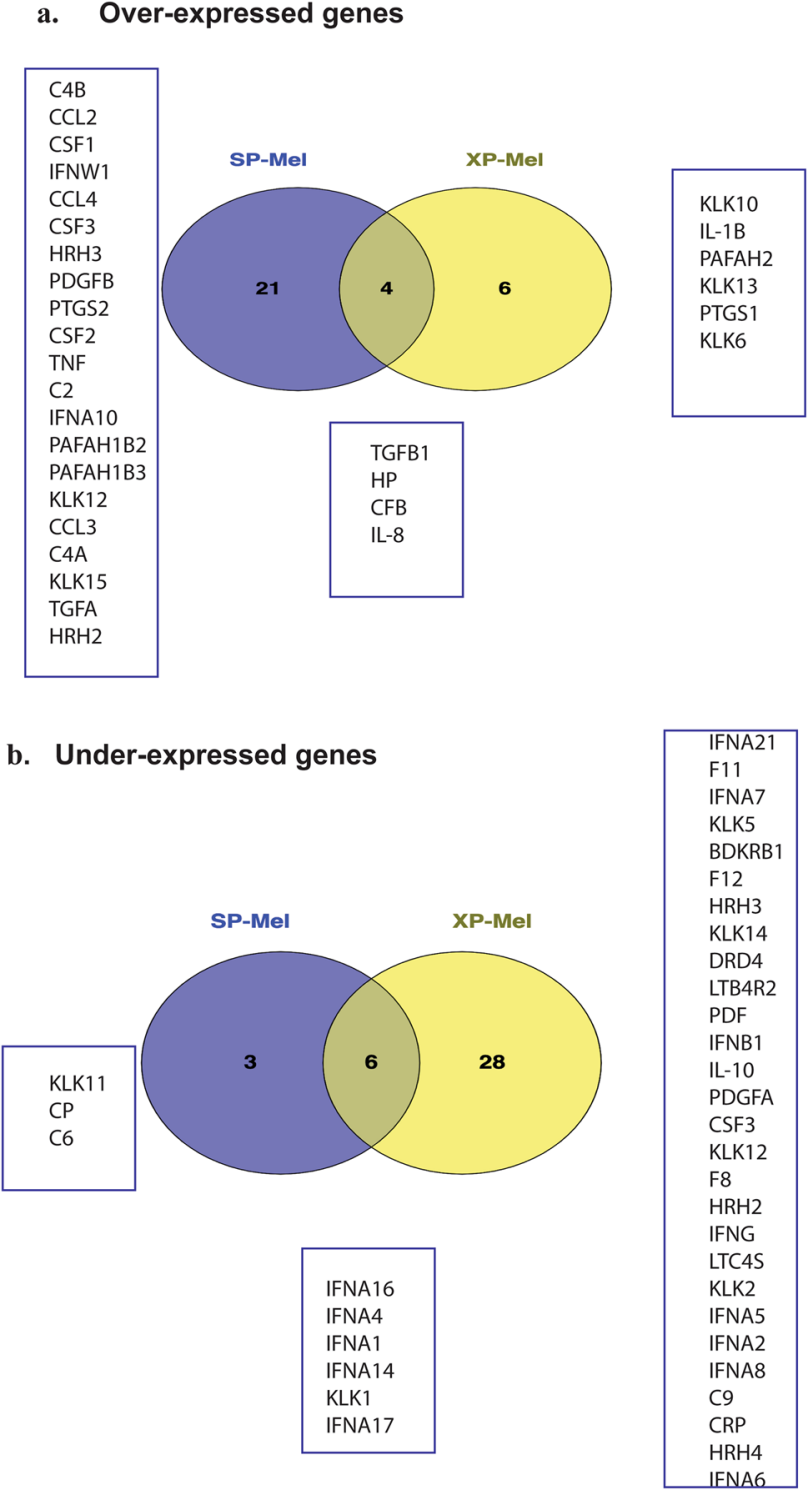
Venn diagram illustrating common and specific differentially expressed inflammatory genes in both groups XP-Mel and SP-Mel compared to healthy control. (**a**) over-expressed genes (Fold change > 2) (**b**) under-expressed genes (Fold change < 0.5).

Regarding the under-expressed genes, 34 out of 84 genes in XP-Mel and 9 out of 84 genes in SP-Mel group were found, with 6 genes under-expressed in common. These data suggest the existence of a common altered inflammatory pathway in XP and SP-Mel (Fig. 1).

### Validation of random genes sets from the pre-screening results of PCR array using qPCR

**Figure 2:**
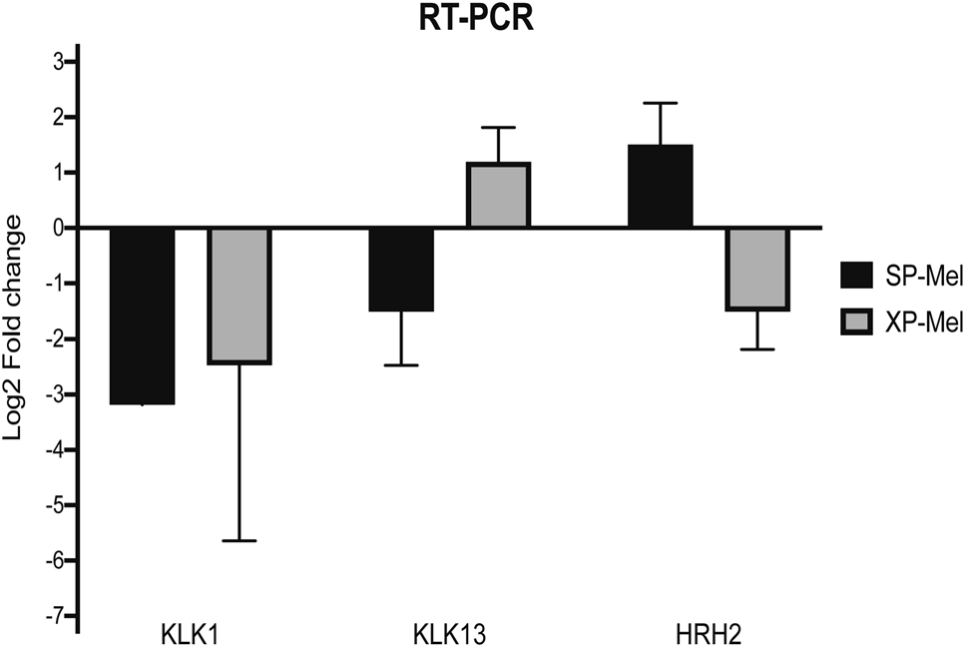
Validation of randomly selected inflammatory genes by RT-qPCR . Data are represented as Log2 fold change. Results are presented as mean ± standard error for RT-qPCR normalized to 2 housekeeping genes RPLP0 and PPIA (mean of triplicate analyses for XP-Mel (n = 3) and SP-Mel (n = 3).

Primers were designed for 3 randomly selected genes identified by PCR array, whose expression was taken into consideration (2 < fold change < 0.5) in both groups (*KLK1*), or specifically in XP-Mel (*KLK13*), or SP-Mel (*HRH2*) groups. Quantitative real-time PCR (q-PCR) was carried out in triplicate on the same samples as used for the PCR arrays and expression was normalized to healthy skin controls. Results obtained using real-time qPCR were similar to those obtained by PCR array (Fig. 2).

### Investigation of commonly altered (over or under expressed) inflammatory genes expression in PCR array and RNA-seq database

In order to explore the involvement of the commonly expressed inflammatory genes in XP-Mel and SP-Mel, we evaluated their expression in 461 samples of cutaneous melanoma that are included in the cancer genome atlas database (TGCA)^29^. In detail, we consulted the gene expression interactive analysis (GEPIA) online server that combines the data of TGCA, comparing the sporadic melanoma samples with those of 558 matched healthy skin from Genotype-Tissue Expression (GTEx) data sets^30^. Analysis of the different boxplots showed that the results obtained for *TGFB1, IL-8, and KLK1* have the same trend as those obtained by PCR array. In particular, statistically significant over-expression of *IL-8* was noted in cutaneous melanoma (TPM = 0.27 in healthy skin compared to melanoma tumors TPM = 4. 52 (adjusted *p*-value 6.39e−99 ); and a significant under-expression was obtained for *KLK1* (TPM = 70.74 in healthy skin compared to Melanoma tumors TPM = 0.52 (adjusted *p*-value0.00e + 0). For the *IFNA* gene family, their expression was too low to be considered in both types of tissue (TPM < 10).

**Figure 3:**
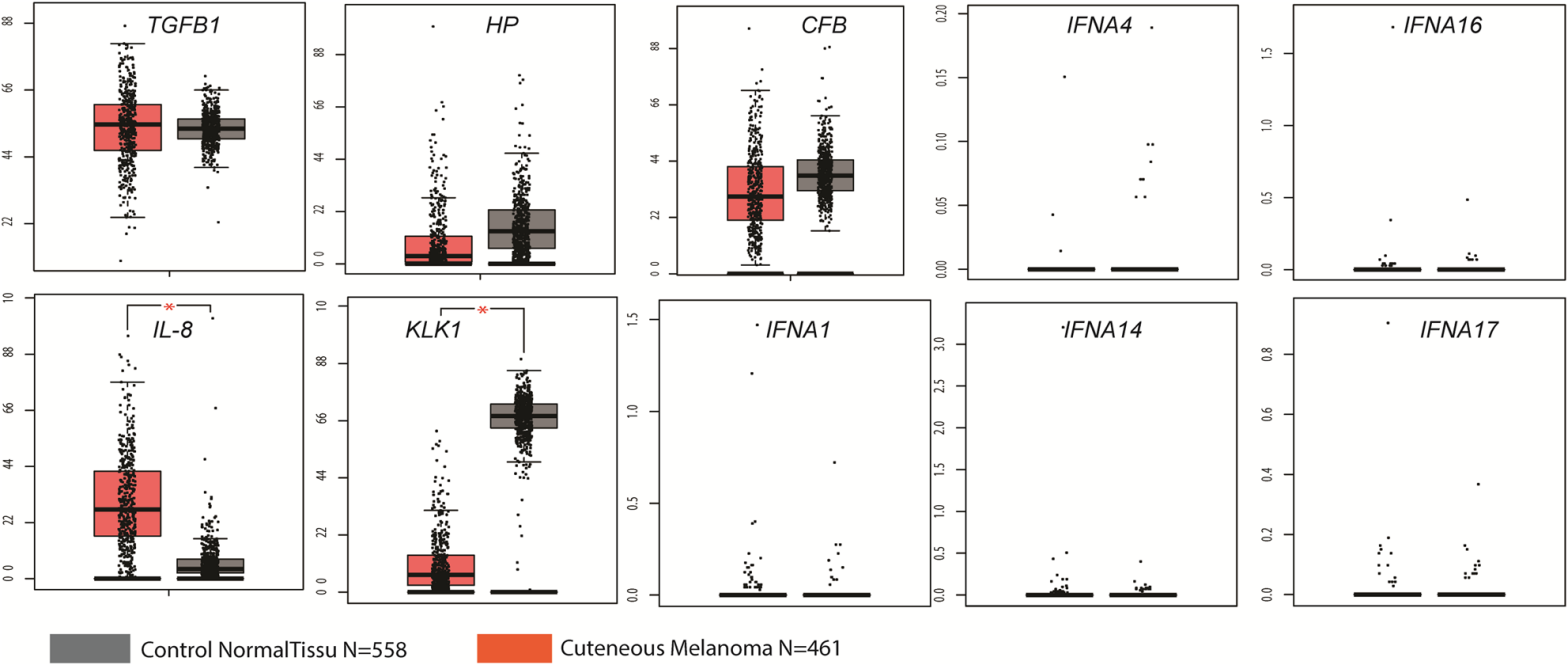
Exploration of 10 commonly expressed inflammatory genes in XP-Mel and SP-Mel via RNA-seq data of sporadic melanoma using GEPIA web server based on the TCGA and GTEx databases. Box plots represent the gene expression level in terms of log2 (TPM) in tumors (red, n = 461) and healthy skin (grey, n = 558) samples, respectively. Healthy skin is matched TCGA adjacent tissue and GTEx data. The method for differential analysis is one-way ANOVA (*p-value < 0.05).

Two genes namely *HP and CFB* have different expression profiles compared to the results found using PCR array (Fig. 3).

### Impact of sun sensitivity on the differentially expressed inflammatory genes in XP-Mel samples

**Figure 4:**
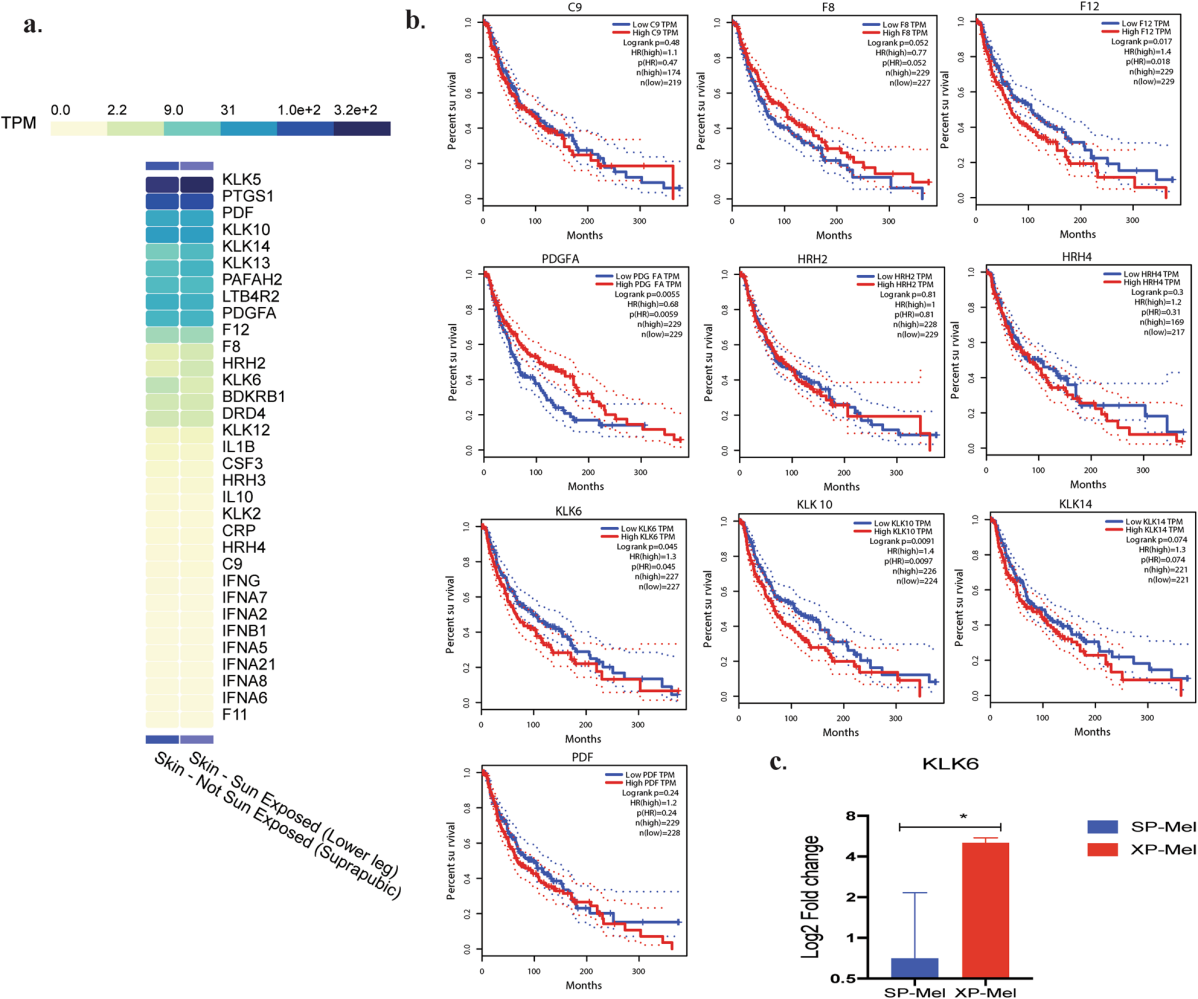
Exploration of the 34 specific inflammatory genes in XP-Mel tumors in GTEx and GEPIA databases: (**a**) Heatmap depicting the altered inflammatory genes via GTEx platform in healthy and sun-exposed skin samples; results are expressed in TPM (the number of Transcripts Per million Readings). (**b**) Overall survival outcomes of patients with sporadic melanoma with low and high gene expression of *C9, F8, F12, PDGFA, HRH2, HRH4, KLK6, KLK10, KLK14, and PDF*. Kaplan–Meier curves are plotted using the GEPIA online Tool, 95% confidence intervals are shown (**c**) KLK6 gene expression in SP-Mel and XP-Mel, normalized expression from the PCR array results. Hazard ratios (HR); (*p-value < 0.05).

We explored specifically the 34 differentially expressed genes in the XP-Mel group to assess whether the difference in the expression of the inflammatory genes (XP-Mel versus healthy skin) is due specifically to the accumulation of UVs damage in XP tumors or due to defects in the NER pathway that affects the functioning of the immune system. For this purpose, the RNA-seq transcriptomic data in the GTEx database was explored for the expression of the 34 genes in skin exposed to solar radiation compared to non-exposed skin. Nine out of 34 genes (*PTGS1, DRD4, CSF3, PAFAH2, BDKRB1, HRH3, LTB4R2, KLK13*, KLK2) have the same expression trend as the results found using the PCR array in XP-Mel, which may be linked to the extreme sun sensitivity in these patients. While the 13 other genes were not detected or were not taken into account due to unavailable data in GTEx database (Fig. 4a).

As for the 12 genes whose expression in RNA-seq was different from the PCR array data (*KLK10, KLK6, HRH4, C9, F8, F12, PDGFA, HRH2, KLK14, PDF, KLK5, KLK12*), we speculate that their altered expression was related to the bad survival rate upon tumor development in XP patients. We, therefore, explored the GEPIA webserver to estimate how the level of expression for 10 of these genes was associated with the overall survival (OS) in sporadic forms (OS: time from cancer diagnosis to death for any cause). Kaplan–Meier analysis was used to compare between the subgroups with high and low gene expression (using the median, 50% quartile values of gene expression as cut-off points) in a cohort of patients with sporadic cutaneous melanoma, which was available in GEPIA database. The OS outcome was significantly associated with a high level of *PDGFA* and a low level of *KLK6* and *KLK10* expression in melanoma tumors (*p*-value = 0.0055, *p*-value = 0.045, and *p*-value = 0.0091, respectively). These genes were expressed in an opposite way in XP patients who developed melanoma at a young age (Fig. 4b).

It is interesting to note that only the *KLK6* was significantly over-expressed in XP-Mel group according to the results of the PCR array (fold change 5 ± 0.5 *p*-value = 0.03) (Fig. 4c).

### Phenotyping of monocyte subsets in sporadic melanoma and in XP patients

Flow cytometry analysis was performed to assess monocyte phenotypes, in the PBMC of 16 healthy donors (controls), 8 patients with sporadic melanoma (SP-Mel), and 6 XP (3 XP-C, 2XP-A and one XP-V) patients that did not develop cutaneous tumors at the time of the study.

**Figure 5:**
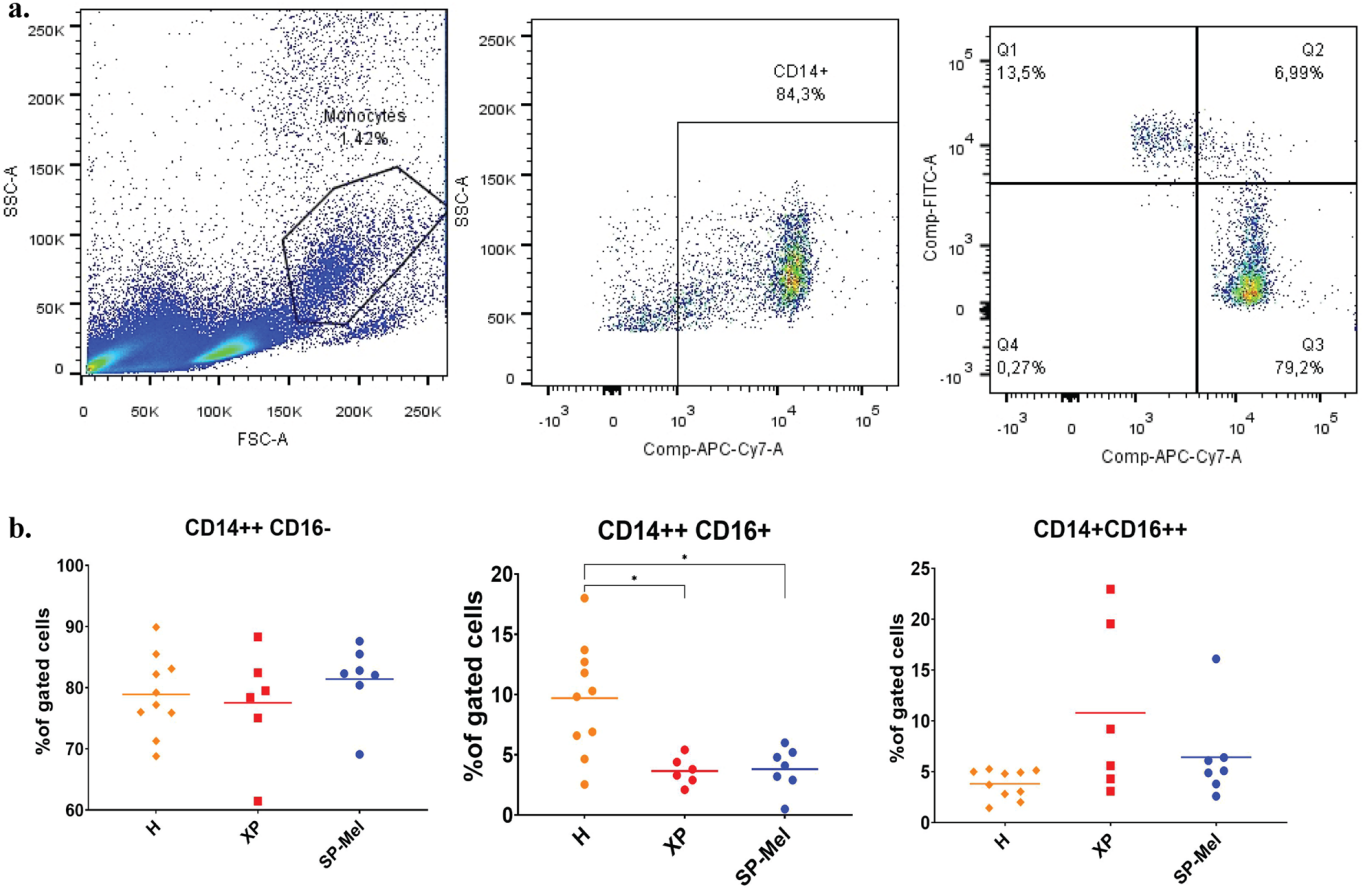
Characterization of blood monocyte’s subsets in healthy donors (n = 18), in patients with sporadic melanoma SP-Mel (n = 8), and in Xeroderma Pigmentosum patients (n = 6) (**a**) Gating strategy for the identification of the three monocyte’s subsets (**b**) Percentages of the different monocyte’s subsets CD14 +  + CD16- classical CD14 + CD16 + intermediate and CS14 + CD16 +  + non classical (*p-value < 0.05).

For classical monocytes, with a CD14 +  + CD16- phenotype, there was no difference in both groups compared to healthy donors 80% ± 2. For intermediate monocytes, with CD14 +  + CD16 + phenotype, there was a significant decrease in their percentage in patients with SP-Mel 3.8% ± 1.82 (*p*-value = 0.009) and in XP patients 3.6% ± 1.16 (*p*-value = 0.01) compared to healthy controls 9.7% ± 4.6. As for non-classical monocytes with a CD14 + CD16 +  + phenotype, there was no significant difference in the three groups SP-Mel 6.42% ± 4.45, XP 10.78% ± 8.4 compared to healthy controls 3.8% ± 1.4 (Fig. 5).

## Discussion

Melanoma is a rare form of skin cancer especially in the Tunisian population^1^. It is the source of high mortality rates^1^. Sun exposure and UVs radiation are well-established risk factors. The exceptionally high incidence and early onset of melanoma in patients with XP indicate that DNA repair is involved in the etiology of melanoma. Advances in high-performance technology make it possible to test individuals at-risk for melanoma, leading to the discovery of new preventive methods in the general population, such as adequate use of sun protection and skin cancer screening at regular intervals, as well as the use of chemo-preventative agents^31^.

Skin cancer develops early in XP patients in whom the risk increases 10,000 times compared to the general population as in the case of the United States where XP patients develop melanoma at an average age of 22 years old^32^. Although North African XP patients develop mostly basal cell carcinomas, and more rarely melanomas, and spinocellular carcinoma^33^ we noted that the XP patients develop melanoma 42 years earlier than sporadic cases. In this work, the three Tunisian XP patients developed cutaneous melanoma at an average age of 17 years, which was in accordance with a previous study conducted in the Tunisian population^1^ . It is important also to note that this study was done through 5 years of monitoring in collaboration with the dermatology department and that we were able to reach only 3 XP patients who developed Melanoma. Extensive patient awareness of the harmful effects of UV exposure has reduced the number of skin cancers in XP patients and in particular melanoma^4^. This work includes two patients from XP-C complementation group and one XP-A.

High inflammation level observed via Hematoxylin and Eosin staining on paraffin section from the XP-A patient (data not shown) may be associated to the severe sunburn reaction observed in these patients^34^. XP-A and XP-C patients develop skin cancers with a similar average age ranging from 8 to 20 years old^4^ .

Although XP-A patients develop also neurological damage, they exhibit the same cutaneous histological manifestations. In our study, the expression of inflammatory genes was made only from cutaneous melanoma.

The mechanisms involved in the development of skin cancers in XP patients have been linked solely to the impact of DNA repair defects following exposure to UVs in most of the studies. However, in other DNA repair syndromes with similar underlying pathways such as Fanconi anemia and Bloom syndrome, investigations suggested an impact of the DNA repair impairment on the immune system. Indeed, patients with Bloom syndrome have low number of lymphocytes, with mild immunodeficiency affecting the class switch recombination process during B cell development^11^. While other reports on patients with Fanconi anemia, reported low NK-cell number and altered functions as well as a decrease in cytotoxic T-cell responses^10,35^.

Cancer susceptibility in XP patients is not solely due to persistent DNA lesions due to defective DNA repair after UV exposure, but could be the result of a more complex situation. In fact, XP patients protected from UVs exposure develop other internal cancers such as myeloid leukemia^4,36,37^ and the cause behind this is still unknown.

Recent studies highlighted the possibility of oxidative stress involvement in the development of cancers in DNA repair diseases, and in age-related pathologies^38^. This could influence immune responses. XP patients represent a form of skin aging even in mild forms^39^ , hence our hypothesis about the involvement of immunity and particularly inflammatory actors in the pathophysiology of skin cancer in XP patients.

As XP is a rare disease, few studies investigated the immune responses. Indeed, following clinical observations, several immunological defects have been reported in XP patients, including the reduced lytic activity of NK cells^40^, decreased production of interferons by lymphocytes^41^, decreased proportion of TCD4, and TCD8 lymphocytes^42^, as well as altered UV-induced cytokine production^43^.

More recently, studies of the tumor microenvironment of Basal cell carcinoma in XP patients also showed increased expression of apoptosis-related biomarkers in immune cells such as CD95, Bcl-2 and Bax compared to sporadic cases, which may explain the earlier establishment of cancer in these patients^44^. The most recent report also describes a dense infiltration of CD163 + macrophages with anti-inflammatory action, in the tumor microenvironment of basal cell carcinoma^45^.

The analysis of the inflammatory genes’ expression profiles in cutaneous melanoma of XP-Mel and SP-Mel shows a specific transcriptomic signature for each group. The singularities in gene expression patterns in XP-Mel and in SP-Mel have been previously found in BRAF and PTEN pathways, where it has been shown an increased frequency of PTEN mutations and activation of the mTOR pathway in the XP-Mel tumors compared to a lower frequency of BRAF mutations^46^. Several studies have focused on a large-scale analysis of inflammatory genes in cutaneous melanoma^47^, but none has addressed this aspect in XP patients.

PCR array is becoming one of the standard approaches measuring differential gene expression in many diseases such as cancer^48^ and autoimmune diseases^49^. This work represents the first report in the study of a rare genetic disease using pathway-focused PCR-based arrays for candidate genes involved in the immune response and inflammatory process. Genome-wide studies and microarray investigation could be a better strategy to study a more important set of genes, however, it represents low sensitivity for under-expressed genes^50^, like what we noted in the XP-Mel patients (34 out of 84 genes).

DNA damages caused by the UVs in skin cells activate several transcription factors^51^. Although it is well recognized that these changes can cause a shift in gene expression, transcriptional regulation following DNA damage is still poorly understood. Few reports suggest that UV-induced lesions in the transcribed strand of DNA form an obstacle to the RNA Polymerase activity translocation leading to its arrest which induces the activation of nucleotide excision repair system^52^. It is not then surprising that due to XP genetic defects, we found that the expression of some inflammatory genes appears to be altered in melanoma tumors compared to those of sporadic origin.

The selected candidate genes used in the PCR array experiments were mostly inflammatory mediators that are normally released upon UV-radiation of the skin, such as cytokines (e.g. IL-1a, IL-6, IL-8, TNF-alpha), growth factors (e.g. TGF-beta, VEGF, NGF) and vasoactive amines (e.g. histamine, bradykinin,)^53^. Following the PCR array experiments, we observed a higher number of inflammatory genes whose expression was altered in XP-Mel patients (51.16% of total). Indeed, the 34 inflammatory genes were under-expressed in XP-Mel tumors while only 9 were under-expressed in SP-Mel ones. This seems to be associated with an immunodeficiency status in these patients^54^.

Given the rarity of samples used in this study and the lack of data investigating the inflammatory response in XP patients, we consolidate the results via the consultation of larger sampling in melanoma patients through RNA-seq data exploring different databases. One of the common biomarkers in SP-Mel and XP-Mel implicate two genes namely *IL-8* and *KLK1*. *IL-8*, also known as *CXCL8*, was one of the most expressed genes in the studied melanoma samples. This cytokine is not produced by healthy melanocytes and its overexpression in seems contributing to melanoma progression^55,56^.

As for tissue *KLK1*, it exerts a role in producing vasoactive kinins, which in itself carry out a wide range of biological activities, including vasodilation, lowering blood pressure, pain induction, and inflammation^57^. *KLK1* expression was downregulated in both pancreatic and colon cancer in previous studies^58^. However, it was never linked to melanoma. In this work, we noted a low level of *KLK1* expression in both XP-Mel and SP-Mel tumors as well as in RNA-Seq data. These findings require further investigations to determine its role in skin cancer development.

In addition, two genes *HP* (Haptoglobin) and *CFB* (Complement Factor B) had a different expression depending on the technique used, either PCR array or RNA-seq. Regarding *HP*, it is known for its systemic anti-inflammatory function. *HP* affects immune cells and its expression is increased in the mouse model of melanoma^59^, which is in accordance with our PCR array findings. As for *CFB*, little is known about its implication in skin cancers and in particular in melanoma^60^. The variable expression rate of these two genes could be due to the fact that the inflammatory status of the microenvironment of the melanoma tumors that we analyzed is different from that of the samples analyzed via RNA seq.

The analysis of inflammatory gene expression in RNA-seq database GTEx of healthy skin exposed to sunlight revealed that nine genes (*PTGS1, DRD4, CSF3, PAFAH2, KLK13, BDKRB1, HRH3, LTB4R2, and KLK2*) present the same expression profiles as for XP patients. As it is well established, XP patients exhibit extreme sensitivity to sunlight, triggering severe sunburn^61^. These genes are implicated in pathways of prostaglandins, bradykinin and histamine, which are well known as first interactors to induce inflammatory response during sunburn^62^. As for the genes related to the kallekrein families (KLK), it may represent potential targets for studies on severe sunburns.

In this work, we observed that the overall survival outcome was significantly associated with a high level of *PDGFA* and a low level of *KLK6* and *KLK10* expression in melanoma tumors and that these genes were expressed in an opposite way in XP patients who developed melanoma at a young age.

The KLKs represent a family of secreted serine proteases composed mainly of 15 genes. The peptidases of the kallikrein family (KLK) are proteases secreted by granular keratinocytes in the upper layer of the skin; they play an important role in the keratinocyte exfoliation process^63^. They also participate in the degradation of the extracellular matrix and tissue remodeling of many tissues^64^. Atypical KLK expression can disrupt skin barrier homeostasis, resulting in a variety of skin diseases such as Netherton syndrome (NS), atopic dermatitis (AD), rosacea, and psoriasis^65^. However, few studies explored the functionalities of KLKs in cancer initiation and progression^66^.

KLK6 was suggested to be involved in the epidermal proteolytic process that regulates the desquamation process^67^. It is one of the most highly upregulated genes in keratinocytes upon differentiation induction with vitamin D3 analogs^68^. Whereas UV-B rays are essential for endogenous production of vitamin D in the skin and that vitamin D deficiency was generally found in XP patients^69^, its overexpression noted in XP-Mel tumors is therefore unrelated to UVs sensitivity but rather due to tumorigenesis process. In support of this hypothesis, it was shown that *KLK6* is highly expressed in primary melanoma, which points to its involvement in the neoplastic processes and malignant progression^57^. As for *KLK10*, it is expressed in the follicular dendritic cells that are essential for the maturation of B cells which suggest possible implications in the regulation of immune cells in lymphoid tissues^70^. As for the KLK10 gene, we found an overexpression in XP patients. Its increased expression in other types of cancers such as triple-negative breast cancer has been associated with poor prognosis^71^.Giving the Fact that *KLK10* is also expressed in healthy skin^66^, we could also suspect an association with the development of melanoma which would require further exploration.

PDGF (platelet-derived growth factor) is a proangiogenic factor^72^. PDGF-AA (PDGFA), PDGF-BB (PDGFB), PDGF-CC (PDGFC), PDGF-DD (PDGFD), and the PDGF-AB heterodimer (PDGFAB) are the five PDGF ligands known to date^73^. Data of DNA sequencing from different types of tumors indicated that mutations in genes coding for PDGF ligands occur in approximately 20% of melanoma^74^. PDGF-AA is overexpressed in 50% of renal cancer, which is correlated with a good survival rate^75^. Through our study, we also suggest that the under-expression of *PDGFA* is associated with low survival risk in XP patients.

Given the significance of the interferon alpha family genes (IFNA) in the immunotherapy of cutaneous melanoma, we explored their level of expression via qPCR. In fact, IFNA are a group of glycoproteins secreted by immune cells during viral infections and microenvironmental stimuli^76^. It englobes 12 distinct proteins that differ in their specific activities. However, the function of each subset is still not well defined. The lack of *IFNA* genes expression in XP-Mel patients suggests their involvement in the DNA repair process. In accordance with this concept, recent studies suggest that DNA double-strand breaks activate the DNA damage response through *IFNA* expression^77^. Our results therefore suggest that the expression of IFNA subtypes is associated with the NER pathway since the majority of genes associated with the interferon signaling pathway tend to be dysregulated in XP-Mel patients. Moreover, previous work indicates that XP patients have a lack in interferon expression^78^, which is in line with our findings.

For IFNA family genes whose expression level were different or non-existent in the GTEx database, the hypothesis that this alteration is caused by defects in the DNA repair system appears therefore to be plausible. However, we were unable to establish a direct link. This highlights the need for further investigations to explore the link between DNA repair pathways and inflammatory responses.

Peripheral mononuclear cells are the main producer of IFNA^79^. It was suggested that the abundance of the different monocyte subsets in systemic lupus erythematosus is related to the level of expression of different IFN genes in particular, the classical and the intermediate monocytes, which are the main IFN responsive cells^80^. Therefore, the decreased expression of the IFNA genes in the tumor microenvironment could be related to the small number of intermediate monocytes in patients developing melanoma that we observed via flow cytometry.

A distinction between the different monocyte sub-groups in patients with melanoma was performed to evaluate the link with cancer development. In general, intermediate monocytes group (CD14 +  + CD16 +) account for approximately 2–8% of circulating monocytes^25^. We found a major declines of this cell population in patients with melanoma. However, the role of these cells is not well established yet. Several studies mentioned their roles in the production of reactive species of oxygen during the immune response, antigen presentation, the involvement of lymphocyte-T proliferation and activation, inflammatory responses, and angiogenesis^81^. Studying the intermediate monocyte poses many challenges given the fact that no equivalent cells exists in animal models.

A low level of intermediate monocytes was reported in other types of cancers such as squamous cell carcinoma and oropharyngeal cell cancer and was correlated with poor clinical outcome^82,83^. Regarding classical monocytes, it was reported that a decreased number of these cells is associated to cutaneous melanoma stage IV by Chavan et al.^27^, while another study reported an increased number of non-classical monocytes in patients with cutaneous melanoma and associated it with a better response to immunotherapy^28^. The disparity between the results obtained in our work and that reported by Chavan *et al*^27^ can be explained by the fact that our study includes primary melanoma tumors. Moreover, as the intermediate monocytes subtype represents a transitional state between the classical and non-classical subtypes, makes its delineation variable depending on the gating strategies^84–87^.

It is important to note that the XP patients that we have enrolled in the phenotyping of monocytes had not developed cancers at the time of the study. They presented a low rate of intermediate monocytes as in patients with sporadic melanoma, which suggest that this sub-type could be a relevant biomarker for monitoring cancer development.

Although this work is done on a limited number of samples, it is the first study that has focused on the immune landscape in cutaneous melanoma in patients with DNA repair disorders as XP. It is likely that a small sample size could decrease the study's significance level and raises the margin of error, which can contribute to bias as it has been stated in other studies using the PCR array approaches^88^. However, the current research identified the same patterns of gene expression as shown in previous studies that investigated melanoma cancer such as *IL8* and *TGFB*^89^ as well as in the RNA-seq databases that we investigated in this work. Further exploration with larger sample size is required to confirm the current findings and to examine the consequences of the altered transcriptional profile especially concerning *KLK6* and KLK10 who may contribute to the worsening of Melanoma tumorigenesis.

It should also be noted that the gene expression pattern in XP patients could be related to the age differences between this group, the healthy group and the sporadic cases, since the XP patients were young. However, several studies that have conducted inflammatory gene expression have linked this difference to photo-aging of the skin, particularly for the set of genes associated to senescence-associated secretory phenotype (SASP) (IL1b, IL8 , CCL2, CCL3, MCF.)^90^. Moreover, the Xeroderma pigmentosum has recently been considered as a potential model for on premature human aging studies^8^.

Finally, we observed a decrease of intermediate monocytes number in patients with sporadic melanoma and in XP patients that could be considered as a prognostic marker for skin cancer. Further studies exploring the functions of this monocytes subtype in melanoma would be important to investigate.

## Methods

### Patients and healthy donors

The study was carried out in accordance with the Helsinki principles and approved by Institute Pasteur Ethics Committee in Tunisia under the ethical accord number (reference PCI/22/2012/v2).

After obtaining written informed consent from patients (over 18 years old) and from patient’s parents (for minors), melanoma biopsies were obtained from the Department of Dermatology (Hospital Charles Nicolle, Tunis).

Melanoma samples from 3 XP patients (N = 2 XP-C; 1 XP-A forms) and 3 sporadic melanoma tumors were collected for PCR array analysis. For the XP patients, biopsies of 5 mm were taken from the tumors, a half was snap-frozen in liquid nitrogen, and the other part was embedded in paraffin and stained with hematoxylin/eosin to confirm the melanoma diagnosis and for further histological investigations. For healthy skin samples, pieces of tissue (5–8 mm) that are next to seborrheic keratosis or epidermal cysts was obtained from 3 donors. Each recovered healthy skin biopsy was examined by a pathologist following hematoxylin/eosin staining, who verified that there is no inflammatory infiltrate, before proceeding to RNA extraction. More details about the samples we used are described in Table 1.

**Table 1:** Detailed PCR array cohort description

Code	Healthy donors (control)			SP-Mel			XP-Mel		
Age	33	35	40	59	87	72	9	28	16
Sexe	F	M	M	M	M	F	F	F	F
Pathology	Seborrheic keratosis	Epidermal cysts	Seborrheic keratosis	ALM Sporadic melanoma	SSM Sporadic melanoma	ALM Sporadic melanoma	SSM Melanoma in XP patient	LMM Melanoma in XP patient	LMM Melanoma in XP patient
Genetic mutation	NA	NA	NA	NA	NA	NA	XPC V548A fs X572	XPC V548A fs X572	XPA R228X
Localization	Scalp	Face	Face	Left heel	Toe	Foot sole	Cheek	Cheek	Nose
Inflammation level	Low	Low	Low	high	Low	Low	Low	Low	High

NA = Not Applicable.

Blood samples were also collected from 18 healthy donors, 8 patients with sporadic melanoma, and 6 XP patients (3 XP-C, 2 XP-A forms and one XP-V) for flow cytometry analysis.

### PCR array

RNA is isolated from melanoma biopsies and from healthy skin using trizol extraction method via Rnaeasy kit (Qiagen). RNA concentration and purity were determined by using a NanoDrop. Then, 1000 ng of RNA were reverse transcribed to single-stranded cDNA in a total volume of 20 µL, using reverse transcriptase (Superscript II, #18064014, Invitrogen), according to the manufacturer’s protocol.

Analysis of 84 inflammatory gene’s expression, as well as of eight housekeeping genes (HKG), was performed using PCR array for human inflammation signaling pathways (anygenes, #IF1H1), whose gene list is presented in (Supplementary Table S2). The qPCR was done according to the manufacturer’s protocol and Ct values were measured via LightCycler 480 software (Roche). Data analysis of the raw data was carried out using the ∆∆CT method and Ct were normalized to the mean values of three stable HKG (PPIA, ACTB, and RPLP0). Fold-change calculations were done using the Anygenes sign array data analysis excel sheet available online (https://www.anygenes.com/home/resources/data-analysis-tools), which automatically calculates the fold-change in gene expression between the melanoma patients and the healthy donors group.

### qPCR

We tested the expression of *KLK-13*, *HRH2*, *KLK1* genes using the SYBR Green-based qPCR technique. Primers were selected from the Primer bank database (https://pga.mgh.harvard.edu/primerbank/) and ordered from sigma life science (Table 2). Relative quantification Ct values were obtained from the threshold cycle number of a triplicate test and normalized to the healthy skin. *PPIA* and *RLPO* were used as housekeeping genes.

**Table 2:** List of qPCR Primers.

Primer	Sequence 5' to 3'
HRH2-fw	CGTGTCCTTGGCTATCACTGA
HRH2-rw	GGCTGGTGTAGATATTGCAGAAG
KLK1-fw	CCCGATTCAGTCCCGGATTG
KLK1-rw	AGCTGGTAATTGTCGCTGATG
KLK13-fw	TTGGCCTTGTCAGGAGGTG
KLK13-rw	AGGACCCATTTGGGGTGGA

The LightCycler 480 SYBR Green I Master Mix (# 04 707 516 001, Roche) was used according to the supplier’s recommendations. Q-PCR was performed on LightCycler 480 System (Roche Diagnostics). The program consisted of an initial denaturation at 95 °C for 10 min, followed by 45 amplification cycles (95 °C for 10 s, 60 °C for 10 s, and 72 °C for 20 s). This was followed by a melting program of 95 °C for 5 s and 65 °C for 1 min and 97 °C with continuous monitoring of the fluorescence. The final step consisted of cooling at 2.2 °C/s to 40 °C. Threshold cycle (Ct) was used to calculate relative gene expression by the 2-ΔΔCT method.

### Flow cytometry analysis

Blood samples from 18 healthy donors, 8 patients with sporadic melanoma, and 6 XP patients (3 XP-C, 2 XP-A forms and one XP-V) were collected. Written informed consent was obtained from all donors. Peripheral Blood Mononuclear Cells (PBMC) were extracted from heparinized blood samples using density gradient centrifugation on a Ficoll cushion.

Monocyte phenotyping according to surface markers expression was performed using monoclonal antibodies: anti-CD14 labeled with APC-Cy7.5 (clone MP9, # 557831, BD Biosciences) and anti-CD16 labeled with FITC (clone 3G8, # 555406, BD Biosciences). Plot acquisition was done on flow cytometer FACSCanto™ II (BD Biosciences) for at least 10.000 events and analyzed using flowjo software.

### Differential inflammatory gene expression and survival analysis from the GTEx and GEPIA databases in melanoma skin cancer

Expression of common inflammatory genes of interest, identified in the in sporadic melanoma SP-Mel and XP-Mel biopsies using PCR array were further tested in RNA-seq-melanoma dataset (n = 461) with matched control samples (n = 558). Data was acquired from the GEPIA webserver (http://gepia.cancer-pku.cn/).

As for the genes of interest whose expression was specific to XP-Mel patients, it was compared to the GTEx dataset of sun-exposed and non-exposed skin (https://www.gtexportal.org/home/). The XP-Mel genes of interest were also examined in terms of overall survival in Melanoma patients through the GEPIA database.

### Statistical analysis

Differences in the fold change expression between XP-Mel, SP-Mel and healthy controls following qPCR, were tested by the Mann–Whitney U test. The level of significance was set at 0.05 (*p*-value < 0.05). The analyses were performed using GraphPad software version 7.00 for Windows, GraphPad Software, La Jolla California USA, www.graphpad.com.

### Ethical approval

The study was carried out in accordance with the Helsinki principles and approved by Institute Pasteur Ethics Committee in Tunisia under the ethical accord number (reference PCI/22/2012/v2).

## Supplementary Information

Supplementary Information.

## Data Availability

All processed data have been provided in the manuscript. The corresponding author upon reasonable request could provide raw data, generated for this study.
