# Supplementary material for: Inflammatory landscape in Xeroderma pigmentosum patients with cutaneous melanoma
Source: Sci Rep. 2022 Aug 16;12:13854. doi: 10.1038/s41598-022-17928-z (PMC9381529; doi:10.1038/s41598-022-17928-z)
Supplement: Supplementary file 1 — Supplementary Information. [file 41598_2022_17928_MOESM1_ESM.pdf]

Inflammatory landscape in Xeroderma pigmentosum patients with cutaneous melanoma

Asma Chikhaoui[1,2], Meriem Jones[3], Tadeja Režen[4], Melika Ben Ahmed[5], Chokri Naouali[1,2], Radovan Komel[4], Mohamed Zghal[6], Samir Boubaker[1,2], Sonia Abdelhak[1,2], Houda Yacoub-Youssef[1,2*]

1 Laboratoire de Génomique Biomédicale et Oncogénétique (LR16IPT05), Institut Pasteur de Tunis, Tunisie.

2 Université Tunis El Manar, Tunis, Tunisie.

3 Département de Dermatologie, Hôpital Charles Nicolle de Tunis, Tunisie.

4 Centre for Functional genomics and Bio-Chips & Medical Centre for Molecular Biology, Institute of Biochemistry and Molecular Genetics, Faculty of Medicine, University of Ljubljana, Ljubljana, Slovenia.

5 Laboratoire de Transmission, Contrôle et Immunobiologie de l'infection, LR16IPT02, Institut Pasteur de Tunis Université de Tunis El Manar I, 2092 Tunis, Tunisie.

6 Association for the support of children with Xeroderma pigmentosum (enfants de la lune en Tunisie).

**Gene ID**	**Gene Description**	**SP-Mel**		**XP-Mel**	
		**Fold change**	**p-value**	**Fold change**	**p-value**
LTC4S	Leukotriene C4 Synthase	0.60	0.819	**0.24**	0.352
BDKRB1	Bradykinin Receptor B1	1.03	0.601	**0.48**	0.442
C2	Complement C2	**2.66**	0.311	1.50	0.578
C4A	Complement C4a	**3.96**	0.149	0.91	0.665
C4B	Complement C4b	**10.24**	0.170	1.30	0.686

C6	Complement C6	**0.14**	0.375	1.14	0.798
C9	Complement C9	0.69	0.745	**0.14**	0.335
CCL2	chemokine ligand 2	**2.10**	0.229	1.02	0.921
CCL3	chemokine ligand 3	**3.94**	0.374	1.20	0.928
CCL4	Chemokine (C-C motif) ligand 4	**2.29**	0.429	1.22	0.858
CFB	Complement factor B	**4.13**	0.383	**2.43**	0.539
CP	Ceruloplasmin (ferroxidase)	**0.30**	0.247	0.56	0.323
CRP	C-reactive protein (CRP)	1.53	0.651	**0.14**	0.339
CSF1	Colony stimulating factor 1 (macrophage)	**2.19**	0.426	1.18	0.652
CSF2	Colony stimulating factor 2 (granulocyte-macrophage)	**2.50**	0.440	1.08	0.761
CSF3	Colony stimulating factor 3 (granulocyte)	**2.36**	0.429	**0.39**	0.441
DRD4	Dopamine Receptor D4	1.47	0.497	**0.47**	0.463
F11	Coagulation factor XI	1.29	0.533	**0.10**	0.361
F12	Coagulation factor XII (Hageman factor)	1.18	0.550	**0.48**	0.446
F8	Coagulation factor VIII, procoagulant component	1.51	0.721	**0.37**	0.390
HP	Haptoglobin	**3.78**	0.621	**3.35**	0.504
HRH2	Histamine receptor H2	**7.19**	0.164	**0.37**	0.351
HRH3	Histamine receptor H3	**2.40**	0.454	**0.47**	0.524

HRH4	Histamine receptor H4	0.72	0.528	**0.12**	0.345
IFNA1	Interferon, alpha 1	**0.38**	0.600	**0.08**	0.349
IFNA10	Interferon, alpha 10	**2.69**	0.613	0.55	0.397
IFNA2	Interferon, alpha 2	0.51	0.664	**0.15**	0.328
IFNA14	Interferon, alpha 14	**0.36**	0.882	**0.12**	0.324
IFNA16	Interferon, alpha 16	**0.40**	0.688	**0.11**	0.376
IFNA17	Interferon, alpha 17	**0.11**	0.678	**0.31**	0.403
IFNA21	Interferon, alpha 21	0.57	0.566	**0.10**	0.349
IFNA4	Interferon, alpha 4	**0.40**	0.861	**0.12**	0.360
IFNA5	Interferon, alpha 5	1.18	0.559	**0.18**	0.341
IFNA6	Interferon, alpha 6	1.02	0.910	**0.12**	0.345
IFNA7	Interferon, alpha 7	1.40	0.484	**0.08**	0.345
IFNA8	Interferon, alpha 8	0.57	0.628	**0.14**	0.364
IFNB1	Interferon, beta 1, fibroblast	0.62	0.444	**0.41**	0.554
IFNG	Interferon, gamma	1.03	0.725	**0.33**	0.389
IFNW1	Interferon, omega 1	**2.22**	0.895	0.50	0.438
IL-10	Interleukin 10	0.96	0.490	**0.41**	0.382
IL-1B	Interleukin 1, beta	1.32	0.706	**2.25**	0.335

IL-8	Interleukin 8	**9.99**	0.378	**8.42**	0.388
KLK1	Kallikrein 1	**0.27**	0.747	**0.27**	0.371
KLK10	Kallikrein-related peptidase 10	1.23	0.627	**2.03**	0.102
KLK11	Kallikrein-related peptidase 1A	**0.42**	0.157	1.00	0.904
KLK12	Kallikrein-related peptidase 12	**3.47**	0.419	**0.38**	0.463
KLK13	Kallikrein-related peptidase 13	0.66	0.657	**2.49**	0.111
KLK14	Kallikrein-related peptidase 14	0.61	0.480	**0.47**	0.375
KLK15	Kallikrein-related peptidase 15	**4.54**	0.369	1.41	0.642
KLK2	Kallikrein-related peptidase 2	1.12	0.455	**0.22**	0.364
KLK5	Kallikrein-related peptidase 5	1.41	0.364	**0.50**	0.364
KLK6	Kallikrein-related peptidase 6	0.70	0.710	**5.00**	**0.032**
LTB4R2	Leukotriene B4 receptor 2	1.01	0.627	**0.46**	0.418
PAFAH1B2	Platelet-activating factor acetylhydrolase 1b, catalytic subunit 2	**2.78**	0.242	1.26	0.548
PAFAH1B3	Platelet-activating factor acetylhydrolase 1b, catalytic subunit 3	**3.08**	0.391	1.49	0.768
PAFAH2	Platelet-activating factor acetylhydrolase 2	0.81	0.528	**2.45**	0.892
PDF	Peptide deformylase	0.65	0.753	**0.45**	0.272
PDGFA	Platelet-derived growth factor alpha polypeptide	1.14	0.994	**0.40**	0.307
PDGFB	platelet derived growth factor subunit B	**2.43**	0.502	0.85	0.585

PTGS1	Prostaglandin-endoperoxide synthase 1	0.95	0.652	**4.51**	0.578
PTGS2	Prostaglandin-endoperoxide synthase 2	**2.44**	0.375	1.30	0.515
TGFA	Transforming growth factor, alpha	**5.24**	0.309	0.85	0.595
TGFB1	Transforming growth factor, beta 1	**2.31**	0.832	**2.90**	0.505
TNF	Tumor necrosis factor	**2.56**	0.485	0.62	0.600

Supplementary Table S1 : Differential gene expression of inflammatory genes by PCR array (Threshold set as 0,5>fold change>2)

	1	2	3	4	5	6	7	8	9	10	11	12
A	HRH1	HRH2	HRH3	HRH4	KLK1	KLK2	KLK3	KLK4	KLK5	KLK6	KLK7	KLK8
B	KLK9	KLK10	KLK11	KLK12	KLK13	KLK14	KLK15	BDKRB1	BDKRB2	LTB4R	LTB4R2	LTC4S
C	PTGS1	PTGS2	CCL2	CCL3	CCL4	IL-8	IL-1A	IL-1B	IL-6	IL-10	IFNA1	IFNA2
D	IFNA4	IFNA5	IFNA6	IFNA7	IFNA8	IFNA10	IFNA14	IFNA16	IFNA17	IFNA21	IFNAR1	IFNAR2
E	IFNB1	IFNG	IFNW1	TNF	PDGFA	PDGFB	PDGFC	PDGFD	TGFA	TGFB1	TGFB2	TGFB3
F	IGF1	IGF2	CSF2	CSF3	CSF1	C2	C3	C4A	C4B	C6	C9	PAFAH1B1
G	PAFAH1B2	PAFAH1B3	PAFAH2	CRP	CP	CFB	F8	F11	F12	DRD4	PDF	HP
H	PPIA	ACTB	TBP	B2M	RPLP0	HPRT1	TFRC	GUS	POS CONT	POS CONT	NEG CONT	NEG CONT

Supplementary Table S2: AnyGenes Human inflammatory signaling pathway layout; genes within the same color belonging to the same pathway or family
